# MSFN: a multi-omics stacked fusion network for breast cancer survival prediction

**DOI:** 10.3389/fgene.2024.1378809

**Published:** 2024-08-02

**Authors:** Ge Zhang, Chenwei Ma, Chaokun Yan, Huimin Luo, Jianlin Wang, Wenjuan Liang, Junwei Luo

**Affiliations:** ^1^ Academy for Advanced Interdisciplinary Studies, Henan University, Kaifeng, Henan, China; ^2^ School of Computer and Information Engineering, Henan University, Kaifeng, Henan, China; ^3^ Henan Key Laboratory of Big Data Analysis and Processing, Henan University, Kaifeng, Henan, China; ^4^ College of Computer Science and Technology, Henan Polytechnic University, Jiaozuo, Henan, China

**Keywords:** deep learning, breast cancer survival prediction, multi-omics data, residual graph neural network, convolutional neural network, stacking integration

## Abstract

**Introduction:** Developing effective breast cancer survival prediction models is critical to breast cancer prognosis. With the widespread use of next-generation sequencing technologies, numerous studies have focused on survival prediction. However, previous methods predominantly relied on single-omics data, and survival prediction using multi-omics data remains a significant challenge.

**Methods:** In this study, considering the similarity of patients and the relevance of multi-omics data, we propose a novel multi-omics stacked fusion network (MSFN) based on a stacking strategy to predict the survival of breast cancer patients. MSFN first constructs a patient similarity network (PSN) and employs a residual graph neural network (ResGCN) to obtain correlative prognostic information from PSN. Simultaneously, it employs convolutional neural networks (CNNs) to obtain specificity prognostic information from multi-omics data. Finally, MSFN stacks the prognostic information from these networks and feeds into AdaboostRF for survival prediction.

**Results:** Experiments results demonstrated that our method outperformed several state-of-the-art methods, and biologically validated by Kaplan-Meier and t-SNE.

## 1 Introduction

According to the Global Cancer Statistics 2020, 2.26 million new cases of breast cancer were diagnosed in 2020, and the deaths from breast cancer were in the fifth rank of all cancers (Sung et al., 2021). Breast cancer has become the most prevalent cancer in the world (Arnold et al., 2022). Survival prediction is an essential part of cancer prognosis. It aims to predict the survival risk of cancer patients and provide recommendations for pathologists and doctors in treatment (Hagerty et al., 2005). Accurate and reliable survival prediction can provide doctors with scientific guidance and improve the survival rate of patients. More importantly, the survival prediction tools could formulate reasonable treatment strategies for patients, avoid unnecessary pain caused by over-treatment, and improve the quality of life of patients. Meanwhile, it reduces the burden of doctors and avoids the wastage of medical resources (Deepa and Gunavathi, 2022). Therefore, developing accurate and reliable survival prediction methods is vital for the treatment and prognosis of breast cancer.

With the widespread application of next-generation sequencing technologies and the accumulation of medical data on cancers, plenty of survival prediction methods have been developed, including (i) statistical survival analysis methods and (ii) machine learning-based methods. Statistical survival analysis methods such as CoxPH and LogRank test use survival data and a few covariates to predict patient survival (Michaelson et al., 2002). However, these methods are difficult to model and not applicable to analyzing large amounts of data. Machine learning-based methods effectively address these limits of statistical survival analysis methods. Algorithms such as Support Vector Machines (SVM), Random Forest (RF) and Logistic Regression (LR) obtain prognostic features from large amounts of cancer data to predict survival (Xu et al., 2012). However, machine learning-based methods require researchers to perform laborious and complex feature engineering work.

In recent years, deep learning methods have provided scientists with powerful tools for extracting high-quality prognostic information from massive omics data and have been proven effective in survival prediction (LeCun et al., 2015; Deepa and Gunavathi, 2022). For instance, Ching et al. (2018) developed a neural network model Cox-nnet with a Cox regression layer to predict survival using RNA-Seq data. Katzman et al. (2018) proposed DeepSurv to predict patient survival and the effect of covariates on patient survival risk by combining DNN and Cox-PH. However, the human genome is extremely complex, and various factors influence cancer pathogenesis (Lujambio and Lowe, 2012). Multi-omics data contains a wealth of information, providing an unprecedented opportunity to investigate the occurrence and progression from multiple perspectives (Arjmand et al., 2022). But the deep learning survival prediction methods described above are inapplicable to multi-omics data. Therefore, deep learning methods based on multi-omics data have risen to prominence in survival prediction (Herrmann et al., 2021; Kang et al., 2022).

One kind of survival prediction research predicts patients’ survival risk (survival rate) based on their survival time and survival status. For example, Cheerla et al. proposed introducing the COX loss function in the deep learning model to fusion clinical data, gene expression data, microRNA expression data, and WSIs (Whole Slide Images) to predict the survival rate of patients with 20 cancers (Cheerla and Gevaert, 2019). Li et al. (2022) proposed HFBSurv to predict patient survival by employing a factorized bilinear model to fuse gene expression, CNV, and pathology image features step by step. Another survival prediction research predicts the long and short survival of cancer patients. For instance, Sun et al. proposed MDNNMD, a survival prediction model that integrates clinical, CNV, and gene expression data of breast cancer by fusing three DNNs with different weights (Sun et al., 2018). AMDN extracts prognostic features of clinical and gene expression data using NMF matrix decomposition combined with attention mechanisms to predict breast cancer survival (Chen et al., 2019). Subsequently, Arya et al. proposed a stacked integration model STACKED RF to overcome the limitation that MDNNMD requires manual adjustment of fusion weights (Arya and Saha, 2020). In the follow-up research, they introduced a gated attention mechanism into STACKED RF to enhance the prediction performance, named SiGaAtCNN (Arya and Saha, 2021). However, previous survival prediction studies based on multi-omics data focus on extracting prognostic features from various multi-omics data, rather than patient similarity and correlation of multi-omics data.

To address these issues in classification prediction studies of long and short survival, we propose a novel Multi-omics Stacked Fusion Network (MSFN) for breast cancer survival prediction. First, we construct a patient similarity network using multi-omics data. Then, we employ ResGCN to obtain similarity information of patients and correlation information of multi-omics data. Simultaneously, we construct CNNs for each omics data to obtain the specificity information. Finally, we stack the prognostic information from the hidden layers of the networks and utilize AdaboostRF for survival prediction. The superiority of MSFN is to comprehensively consider the specificity information of multi-omics data, the similarity information of patients, and the correlation information of multi-omics data, stacking these information to achieve more accurate and reliable survival prediction. The contributions of this work are summarized as follows:

- We propose a novel multi-omics stacked fusion network framework that comprehensively obtains survival-related information from multi-omics data for survival prediction.
- We integrate multi-omics data with Similarity Network Fusion (SNF) that sufficiently utilizes the similarity between patients and the correlation of multi-omics data to generate a comprehensive patient similarity network.
- We use ResGCN to extract the prognostic information of the patient similarity network, leveraging its residual connectivity to achieve a deeper network structure while effectively addressing gradient vanishing.

## 2 Materials and methods

### 2.1 Datasets and preprocessing

To investigate the performance of our method, we conducted comprehensive and rigorous experiments on the BRCA multi-omics dataset from TCGA (The Cancer Genome Atlas). We obtained this dataset from the UCSC Xena platform (http://xena.ucsc.edu/) and removed samples and features with missing values above 20%. 1048 patient samples were finally selected, each sample contained clinical, gene expression, CNV, and survival data. This is because clinical, gene expression and CNV data are highly associated with cancer occurrence and progression, and they have been used extensively in previous survival prediction studies (Shlien and Malkin, 2009; Li et al., 2017; Kalafi et al., 2019). Then, we divided patients into long-term and short-term survivors using a threshold of 5-year survival, with long-term survivors labeled as 1 and short-term survivors labeled as 0. The overview description of the dataset is shown in Table 1.

**TABLE 1:** Overview of the dataset.

Description	Value
Threshold (years)	5
Total patients	1048
Long-time survivors	248
Short-time survivors	800
Average survival (months)	42.34

For the clinical data, we first removed not reported data and features and samples with more than 20% missing values. Then, we removed irrelevant text descriptions, markers, and years from the clinical data. Subsequently, according to the data processing procedure in the study by Sun et al. (2018); Arya and Saha (2020); Arya and Saha (2021), we screened clinical features such as age, tumor size, and TNM stage, and performed label coding and binarization for the categorical features. Finally, we obtained 33 features as clinical features. Since there were no missing values in the gene expression and CNV data, we only estimated missing values for clinical data. Specifically, we divided the 33 clinical features into 24 discrete-valued features and 9 continuous-valued features. For continuous features, we use the k-Nearest Neighbor algorithm (KNN) for interpolation then normalized them using the min-max normalization with the range set to [0,1] (Troyanskaya et al., 2001; Patro and Sahu, 2015). For the discrete features we used the mode interpolation (García-Laencina et al., 2015). For gene expression data, we also used the max-min normalization for normalization with the range set to [0,1]. For CNV data, we directly use the discretized raw data. The gene expression and CNV data for each patient in the dataset has 60,488 and 19,729 features. This high dimensionality of data leads to the “dimensionality catastrophe” that negatively affects the performance of deep learning methods (Berisha et al., 2021). Therefore, we used the renowned mRMR algorithm for feature selection (Peng et al., 2005). Then, we searched the optimal number of gene expression and CNV features in steps of 100 (Arya and Saha, 2020; Arya and Saha, 2021). Finally, we selected 400 gene expression features, 200 CNV features, and all 33 clinical features as model inputs, as shown in Table 2.

**TABLE 2:** Feature selection.

Data type	Total features	Selected features
Clinical	190	33
Gene Expression	60,488	400
CNV	19,729	200

### 2.2 Methods

**FIGURE 1:**
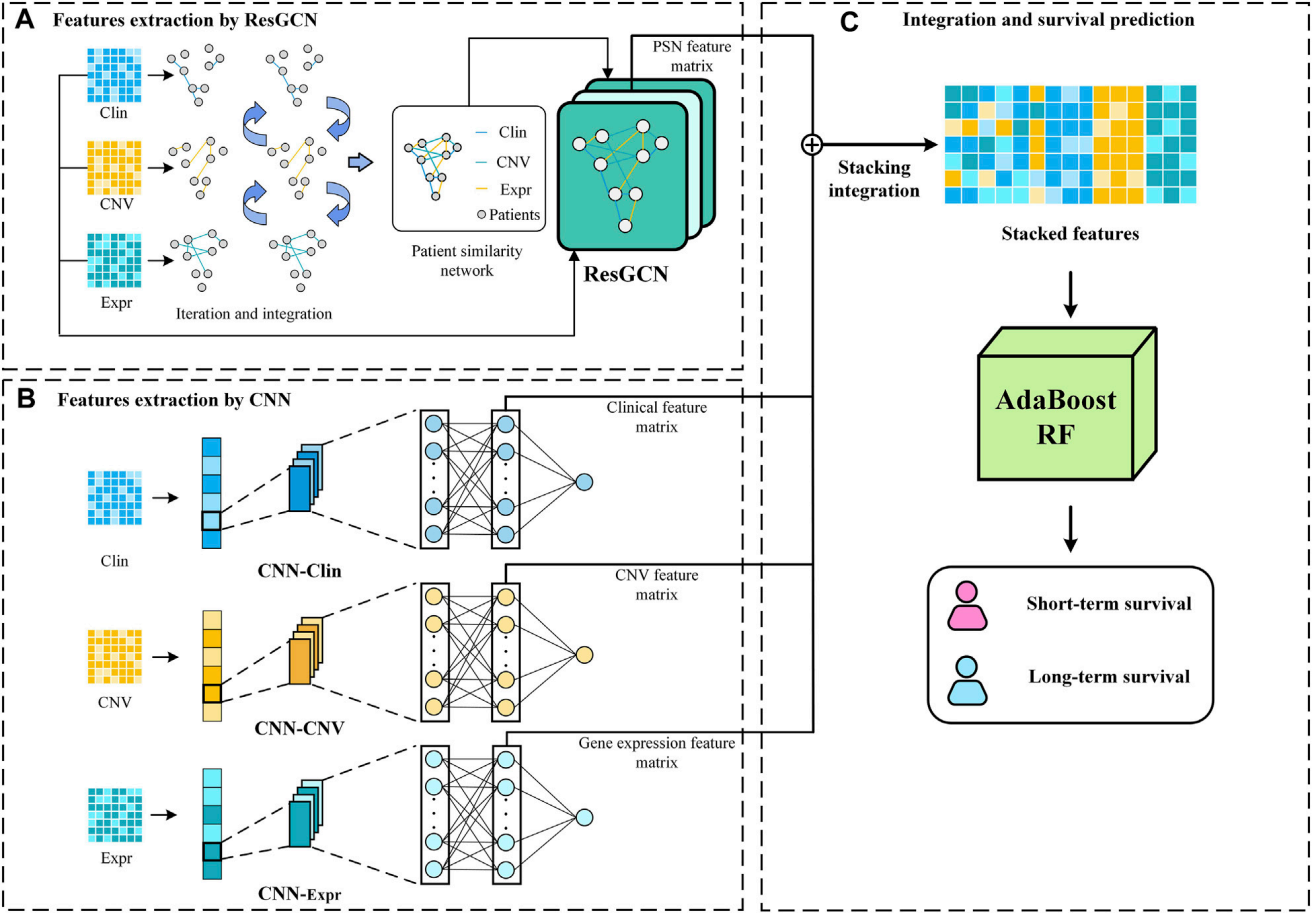
The framework of MSFN.

The proposed MSFN consists of three components. In the first component, MSFN constructs patient similarity networks using SNF and employs ResGCN to obtain similarity information of patients and correlation information of multi-omics data. In the second component, MSFN constructs CNNs for each omics data to obtain the specificity prognostic information. The last component is extracting and stacking the prognostic information of ResGCN and CNNs, feeding them into AdaboostRF for survival prediction. The framework of MSFN is briefly shown in Figure 1. The implementation of our method is available at https://github.com/AckerMuse/MSFN.

#### 2.2.1 A: Features extraction by ResGCN

##### 2.2.1.1 Construction of patient similarity network

In order to construct the patient similarity network, we employ the similarity network fusion (SNF) to construct the patient similarity network (Wang et al., 2014). SNF can integrate multi-omics data from clinical, CNV and gene expression data to generate a comprehensive patient similarity network for fully leverages patients’ similarities and the correlation of multi-omics data. Assuming there are n patients, each patient has m types of data. We denote the patient similarity network as a graph G=(V,E) , where V represents the set of patients,i.e., $\left\{x_{1},x_{2},x_{3},\ldots ,x_{n}\right\}$ . The edge E corresponds to the similarity relation between vertices v∈V in the graph. The weights of these edges are represented by an n×n similarity matrix W , which is computed by Eq. 1:

$$W\left(i,j\right)=\exp\left(-\frac{\vartheta^{2}\left(x_{i},x_{j}\right)}{\lambda \varepsilon_{i,j}}\right)$$ (1)

where λ is the hyperparameter, $\vartheta \left(x_{i},x_{j}\right)$ is the euclidean distance between patients $x_{i}$ and $x_{j}$ , and $\varepsilon_{i,j}$ is used to eliminate the scaling problem (Wang et al., 2014). Then, the similarity matrix is normalized by Eq. 2:

$$P_{i,j}=\left\{\begin{array}{c} \frac{W_{i,j}}{2\sum_{k\neq i}W_{i,k}},j\neq i\\ 1/2,j=i \end{array}\right.\;$$ (2)

Suppose $N_{i}$ is the set of neighbor nodes of $x_{i}$ . We can calculate the similarity matrix L of the single omics data by Eq. 3:

$$L_{i,j}=\left\{\begin{array}{c} \frac{W_{i,j}}{\sum_{k\in N_{i}}W_{j,k}},j\in N_{i}\;\\ 0,otherwise\; \end{array}\right.\;$$ (3)

Let $P_{t}^{\left(v\right)}$ denote the similarity matrix after normalization of the v -th omics data (0<v≤m) in the t -th iteration. Update $P_{t}^{\left(v\right)}$ according to Eq. 4:

$$P_{t+1}^{\left(v\right)}=L^{\left(v\right)}\left(\frac{\sum_{k\neq v}P_{t}^{\left(k\right)}}{m-1}\right){\left(L^{\left(v\right)}\right)}^{T}\;$$ (4)

where the $L^{\left(v\right)}$ denotes the local similarity matrix of the v -th omics data. Through continuous iterative fusion, the SNF ultimately generates a patient similarity network containing correlation information from all omics data. In this work, the patient similarity network is combined with ResGCN for cancer survival prediction.

##### 2.2.1.2 Similarity and correlation features extraction by ResGCN

**FIGURE 2:**
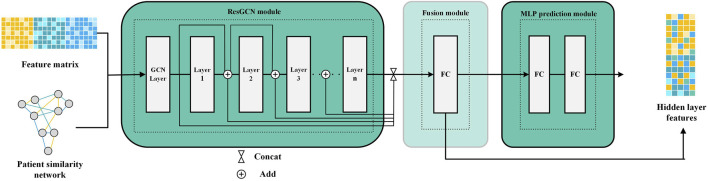
Feature extraction process by ResGCN.

Since the patient similarity network constructed by SNF is graph-structured data, we employ ResGCN to obtain the survival prediction features from it (Li et al., 2019). ResGCN modifies the data transmission mechanism in graph neural networks to mitigate the gradient vanishing problem and overcome the limitation that graph neural networks cannot construct deep networks. As shown in Figure 2, ResGCN takes the feature matrix of multi-omics data and the patient similarity network as input. After the residual graph convolution operation, outputs the feature matrix of the node. The propagation mechanism of ResGCN can be first represented as Eq. 5:

$$G\left(N\right)=f\left(G\left(N-1\right),A\right)=\sigma \left(AG\left(N-1\right)W\left(N\right)\right)\;$$ (5)

where G(N) is the output of the N -th layer, and W(N) is the weight matrix of N -th layer. f() denotes the graph convolution operation. σ() denotes the nonlinear activation function. However, this propagation mechanism only considers the feature vectors of all neighboring nodes, and ignores the nodes themselves. Therefore, self-connection is added to A to overcome this problem, defined as $\hat{A}=A+E$ , where E denotes the identity matrix. Moreover, to avoid changes in the scale of the eigenvectors during the multiplication operation, $D^{-\frac{1}{2}}AD^{-\frac{1}{2}}$ is defined to normalize A , where D is the diagonal node degree matrix. Therefore, the propagation mechanism is redefined as Eq. 6.

$$G\left(N\right)=f\left(G\left(N-1\right)\right)=\sigma \left({\tilde{D}}^{-\frac{1}{2}}\tilde{A}{\tilde{D}}^{-\frac{1}{2}}G\left(N-1\right)W\left(N\right)\right)$$ (6)

Theoretically, deeper networks possess more excellent learning capabilities than shallow neural networks to capture feature representations from more complex data (Bianchini and Scarselli, 2014). Furthermore, deeper neural networks are typically able to achieve outstanding performance with relatively less training data. These are particularly significant for multi-omics data, which are often complex and challenged with limited sample sizes (Picard et al., 2021; Zhang et al., 2021). ResGCN uses residual connections to improve the information flow in the network to alleviate the gradient vanishing problem and allow ResGCN to build deep networks (Li et al., 2019). Thus, the new propagation mechanism can be defined as Eq. 7:

$$G\left(N+1\right)=f\left(G\left(N\right),A\right)+G\left(N\right)=G{\left(N+1\right)}_{\mathrm{res}}+G\left(N\right)\;$$ (7)

After G(N) is transformed by f , vertex-wise addition is performed to obtain G(N+1) . The residual mapping f learns to take the patient similarity network as input and outputs a residual graph representation $G{\left(N+1\right)}_{\mathrm{res}}$ for the next layer. After several layers of residual convolution, the fusion and MLP modules are used to fuse the data processed by multiple residual blocks and output the prediction results. Then, we extract features representing patient similarity information and multi-omics correlation information from the fusion layer in the trained ResGCN. In summary, ResGCN mitigates the gradient vanishing by residual connection mechanism, which improves data transmission in the network and allows for deeper network architecture to fit complex multi-omics data to obtain survival prediction information.

#### 2.2.2 B: Features extraction by CNN

**FIGURE 3:**
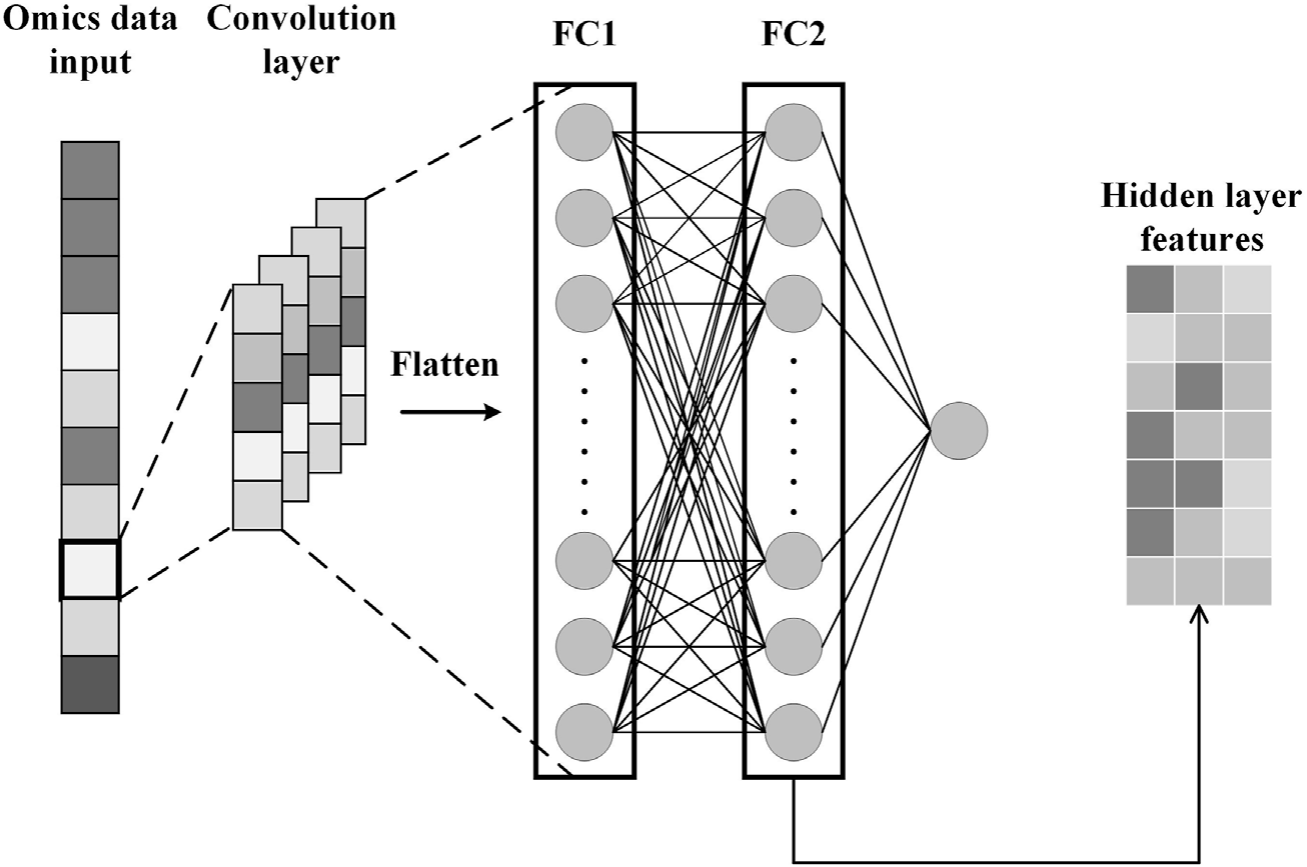
Feature extraction process by CNN.

To obtain specificity features for each omics data, we construct CNN for each omics data. Each CNN consists of an input layer, a convolutional layer, a fully connected layer, and an output layer, as shown in Figure 3. After the omics data is fed into the CNN, the convolution layer performs a convolution operation to generate the feature map and adds padding to the convolutional layer to control the feature map size. Subsequently, the flattening operation maps the output of the convolutional layer to a fully connected layer containing 150 units for survival prediction. In addition, the glorot initialization technique is used to generate random numbers to initialize the convolutional kernel (Glorot and Bengio, 2010). We also applied dropout and L2 regularization techniques to prevent overfitting during training (Cortes et al., 2012; Poernomo and Kang, 2018). Finally, we extract specificity features representing each omics data from the fully connected layers of the three trained CNNs.

#### 2.2.3 C: Stack integration and survival prediction

Stacking hidden layer features of deep learning networks is an effective strategy for integrating multi-omics data for survival prediction (Arya and Saha, 2020; Arya and Saha, 2021). It allows flexible integration of feature representations from different neural network models to integrate correlation prognostic and specificity prognostic information. Moreover, this strategy allows integration in conditions that all neural network modules achieve optimal performance, rather than training all modules simultaneously. We stack the hidden layer features extracted from ResGCN and the three CNNs according to Eq. 8.

$$F_{\mathrm{stacked}}=F_{\mathrm{PSN}}⊕F_{\mathrm{Clin}}⊕F_{\mathrm{Expr}}⊕F_{\mathrm{CNV}}$$ (8)

where $F_{\mathrm{PSN}}$ represents the feature representation obtained from the ResGCN hidden layer, $F_{\mathrm{Clin}},F_{\mathrm{Expr}}$ , and $F_{\mathrm{CNV}}$ represent the feature representation obtained from the CNN hidden layer of each omics data, respectively. $F_{\mathrm{stacked}}$ represents the obtained stacked feature representation, and ⊕ is the matrix concat operation. Then, based on Yifan et al. and Arya et al. we used $F_{\mathrm{stacked}}$ to train the AdaBoostRF for the final breast cancer survival prediction (Arya and Saha, 2020; Yifan et al., 2021).

## 3 Results

### 3.1 Evaluation metrics and experiment settings

To comprehensively evaluate our model, we use the Area Under the Curve (AUC), accuracy, precision, Recall, F1-score, and Matthew’s correlation coefficient (Mcc) as performance evaluation metrics (Goutte and Gaussier, 2005; Huang and Ling, 2005; Chicco and Jurman, 2020). The definitions of these metrics are shown in Eqs 8–14:

$$AUC=\frac{\sum {\left(p_{i},n_{j}\right)}_{p_{i}>n_{j}}}{P\times N}$$ (9)

where P is the number of positive samples. N is the number of negative samples. $p_{i}$ is the positive sample prediction score. $n_{j}$ is the negative sample prediction score.

$$Accuracy=\frac{TP+TN}{TP+TN+FP+FN}\;$$ (10)

$$Precision=\frac{TP}{TP+FP}\;$$ (11)

$$Recall=\frac{TP}{TP+FN}\;$$ (12)

$$F1-Score=\frac{2\times Recall\times Precision}{Recall+Precision}\;$$ (13)

$$Mcc=\frac{TP\times TN-FP\times FN}{\sqrt{\left(TP+FN\right)\times \left(TP+FP\right)\times \left(TN+FN\right)\times \left(TN+FP\right)}}\;$$ (14)

where TP, FP, TN, and FN represent true positives, false positives, true negatives, and false negatives in the confusion matrix, respectively.

To overcome the variance problem caused by the limited sample size and sample imbalance, we used 10-fold cross-validation to evaluate the performance of MSFN (Rodriguez et al., 2009; Jiang and Wang, 2017). The 1048 patients were divided into 10 subsets, 9 of which were combined as the training set while the remaining 1 subset was used as the test set. The final performance was the average of the model’s performance on the test set. MSFN was implemented using Pytorch. The experiments were executed on a PC with a 2.90 GHz Intel Core i7-10700 processor and NVIDIA GeForce RTX 3070 GPU.

### 3.2 Comparison with previous studies

To demonstrate the effectiveness of MSFN. We uniformly used 10-fold cross-validation to evaluate and compare it with several machine learning-based methods and deep learning-based methods. Specifically, we selected three widely used machine learning-based models as the baseline: LR (Logistic Regression) (Jefferson et al., 1997), RF (Random Forest) (Nguyen et al., 2013) and SVM (Support Vector Machine) (Xu et al., 2012). Then, we compared MSFN with five current state-of-the-art deep learning-based models. Below are brief descriptions of deep learning-based methods:

- MDNNMD (Sun et al., 2018): MDNNMD is a DNN-based cancer survival prediction method. It integrates multi-omics data through multiple DNNs and predicts breast cancer survival by setting different weights for network fusion.
- Stacked RF (Arya and Saha, 2020): Stacked RF is a CNN-based cancer survival prediction method. It trains RF to predict breast cancer survival by stacking three CNN networks’ hidden layer feature representations.
- SiGaAtCNN RF (Arya and Saha, 2021): SiGaAtCNN RF is an improved method of Stacked RF. It introduces the gated attention mechanism for better feature representation and stacks hidden layer feature representations of gated attention CNNs for training RF to predict breast cancer survival.
- PregGAN (Zhang et al., 2022): PregGAN is a CGAN-based survival prediction method. It generates high-quality pseudo-samples based on limited samples for reliable survival prediction.
- Heterogeneous stacked RF (Jadoon et al., 2023): Heterogeneous stacked RF is a heterogeneous ensembled classification prediction model that integrates CNN and DNN to predict breast cancer patient survival.

The prediction results are shown in Table 3. From the results, MSFN achieves AUC value of 0.9787 and accuracy of 0.991, which is superior to other methods. Other evaluation metrics are also obviously improved. Specifically, MSFN achieves superior prediction performance compared to SiGaAtCNN RF, Stacked RF, Heterogeneous stacked RF, and MDNNMD because the patient similarity information and multi-omics data correlation information from the patient similarity network provide more comprehensive and wealthy prognostic information for survival prediction. MSFN achieves significant performance improvement compared to traditional machine learning methods and PregGAN which directly integrate multi-omics data. This demonstrates the effectiveness and superiority of the stacked integration strategy in multi-omics data fusion compared to direct data integration.

**TABLE 3:** Performance comparison of MSFN and comparison methods.

Methods	Accuracy	AUC	Precision	Recall	F1-score	Mcc
LR	0.837	0.788	0.548	0.707	0.615	0.523
RF	0.803	0.736	0.452	0.629	0.521	0.413
SVM	0.821	0.757	0.613	0.633	0.618	0.506
MDNNMD	0.697	0.736	0.313	0.233	0.267	0.128
PregGAN	0.814	0.756	0.617	0.580	0.597	0.477
Stacked RF	0.905	0.956	0.831	0.754	0.790	0.731
SiGaAtCNN RF	0.943	0.981	0.873	0.891	0.882	0.845
Heterogeneous stacked RF	0.891	0.826	0.807	0.695	0.758	0.688
MSFN	**0.978**	**0.991**	**0.932**	**0.964**	**0.944**	**0.930**

Bold values represent the highest performance of the model on this metric in the experiment.

### 3.3 Performance comparison of different survival cohorts

**FIGURE 4:**
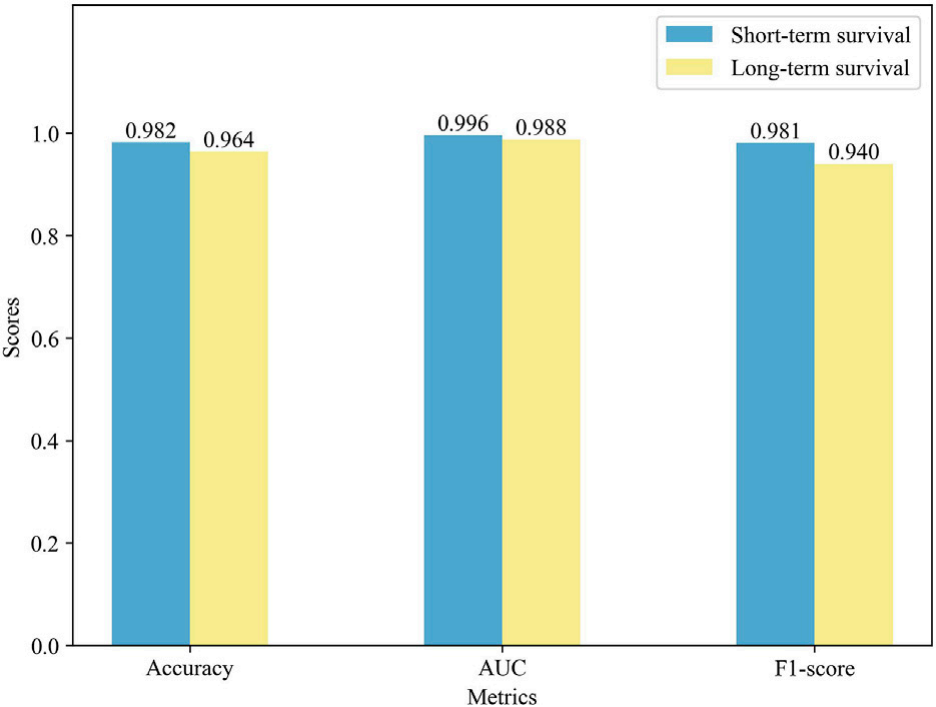
Impact of different ResGCN layers on the model performance.

To further validate the prediction performance of MSFN, we compared its performance in different survival cohorts. We used the ten-fold cross-validation for the experiments and displayed the results in Figure 4. It is obvious that MSFN presents a better prediction performance in both long and short survival cohorts, and the gap between the prediction performance of the two cohorts is very small. This is attributed to that MSFN incorporates the prediction information from different deep learning modules, considering both correlation prognostic information and specificity prognostic information.

### 3.4 Ablation study

We verify how different modules of MSFN affect the performance through an ablation study and design three variants: i) MSFN/-CNNs: MSFN without CNN modules. ii) MSFN/-ResGCN: MSFN without ResGCN module. iii) MSFN/-RF: MSFN without AdaboostRF, and the prediction results are output by MLP. We compared MSFN with the variants described above. As can be seen from Table 4, both MSFN/-ResGCN and MSFN/-CNN perform lower than MSFN. Such results can be attributed to the incomplete prediction features obtained by MSFN/-ResGCN and MSFN/-CNN. This also reflects the importance of integrating prognostic information. Furthermore, MSFN performs better than MSFN/-RF. This is because the AdaboostRF is an ensemble machine learning algorithm with better feature learning ability than simple MLP and effectively deals with complex feature representations of multi-omics data.

**TABLE 4:** Performance comparison between different variants of MSFN.

	Accuracy	AUC	Precision	Recall	F1-score	Mcc
MSFN/-ResGCN	0.902	0.964	0.869	0.735	0.770	0.726
MSFN/-CNNs	0.960	0.975	0.924	0.932	0.928	0.904
MSFN/-RF	0.965	0.951	0.921	0.909	0.937	0.919
MSFN	**0.978**	**0.991**	**0.932**	**0.964**	**0.944**	**0.930**

Bold values represent the highest performance of the model on this metric in the experiment.

### 3.5 Effect of multi-omics data

To validate the effect of multi-omics data, we constructed MSFN using each omics data, respectively. Then, we compared them with MSFN constructed using multi-omics data. As shown in Table 5, Clin, CNV, and Expr represent the MSFN constructed with clinical, CNV, or gene expression data, respectively. The accuracy and AUC only reach a maximum of 0.919 and 0.954 when using single-omics data. MSFN achieves the best performance with multi-omics data, with all evaluation metrics significantly better than single-omics data. This indicates that MSFN can obtain comprehensive prognostic information from multi-omics data and significantly improve prediction performance.

**TABLE 5:** Performance comparison of different omics data.

Data type	Accuracy	AUC	Precision	Recall	F1-score	Mcc
Clin	0.919	0.954	0.863	0.819	0.834	0.787
CNV	0.765	0.770	0.464	0.438	0.431	0.272
Expr	0.804	0.827	0.658	0.407	0.481	0.371
Multi-omics	**0.978**	**0.991**	**0.932**	**0.964**	**0.944**	**0.930**

Bold values represent the highest performance of the model on this metric in the experiment.

### 3.6 Effect of ResGCN layers

**FIGURE 5:**
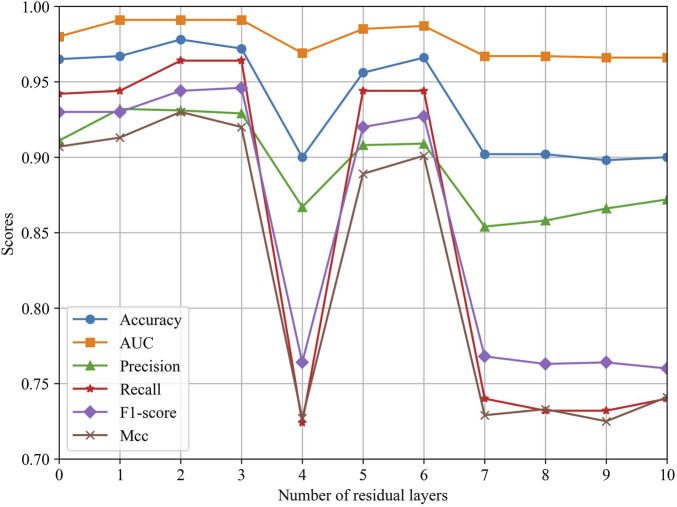
Impact of different ResGCN layers on the model performance.

To explore the effect of different ResGCN layers on the model performance, we evaluated the performance of MSFN by changing the layers of ResGCN. As can be seen in Figure 5, the performance of MSFN gradually improves as the number of ResGCN layers increases. Several metrics achieved their maximum when the layer is set to 2. This demonstrates that the deep ResGCN constructed by residual concatenation can properly fit the multi-omics data, bringing performance improvement to the entire model. However, all metrics fluctuate and gradually decrease as the number of layers increases. This may be because ResGCN with too many layers makes the model structure too complex, leading to overfitting of the model during training.

### 3.7 Effect of the number of features

To explore the effect of the number of features on model performance, we used the incremental method based on previous studies to conduct experiments. Specifically, We employed the mRMR algorithm to select the top 500 features from CNV and gene expression data. Then, we searched with a step size of 100 to evaluate the performance of MSFN under different combinations of feature numbers (Sun et al., 2018; Arya and Saha, 2020). Since only 33 features were available in clinical data, we used all the clinical features. The final results are presented in Table 6. It is evident that the model’s performance gradually improves as the number of features increases. MSFN achieves the best accuracy, AUC, and Recall when the number of CNV and gene expression features is set to 200 and 400. However, as the number of features increases, the model’s performance remains relatively stable and then gradually decreases. This shows that too many features inevitably introduce noisy information, reducing the model’s focus on valuable features and leading to performance degradation. Consequently, we selected the top 200 CNV and top 400 gene expression features along with all 33 clinical features as model inputs.

**TABLE 6:** The results of incremental feature number selection.

CNV	Gene expression	Accuracy	AUC	Precision	Recall	F1-score	Mcc
100	100	0.892	0.970	0.853	0.740	0.760	0.709
100	200	0.968	0.989	0.904	0.908	0.910	0.925
100	300	0.960	0.980	0.927	0.948	0.951	**0.949**
100	400	0.962	0.984	0.906	0.936	0.919	0.894
100	500	0.966	0.985	0.932	0.962	0.950	0.928
200	100	0.969	0.990	0.931	0.936	0.946	0.923
200	200	0.962	0.989	0.931	0.960	0.941	0.929
200	300	0.951	0.983	0.896	0.924	0.908	0.88
200	400	**0.978**	**0.991**	0.932	**0.964**	0.944	0.930
200	500	0.971	0.990	0.932	0.960	0.943	0.930
300	100	0.956	0.979	**0.944**	0.960	0.940	0.934
300	200	0.950	0.988	0.916	0.888	0.899	0.873
300	300	0.960	0.987	0.942	0.956	0.948	0.931
300	400	0.965	0.991	0.937	0.954	**0.951**	0.928
300	500	0.971	0.990	0.929	0.952	0.947	0.930
400	100	0.960	0.987	0.950	0.960	0.948	0.946
400	200	0.949	0.982	0.889	0.920	0.896	0.883
400	300	0.973	0.989	0.940	0.952	0.948	0.933
400	400	0.974	0.991	0.931	0.961	0.939	0.926
400	500	0.904	0.972	0.870	0.744	0.777	0.731
500	100	0.960	0.989	0.935	0.960	0.938	0.946
500	200	0.971	0.991	0.931	0.956	0.934	0.923
500	300	0.912	0.976	0.890	0.740	0.785	0.753
500	400	0.972	0.987	0.935	0.964	0.950	0.918
500	500	0.960	0.992	0.924	0.924	0.914	0.900

Bold values represent the highest performance of the model on this metric in the experiment.

### 3.8 Survival analysis

**FIGURE 6:**
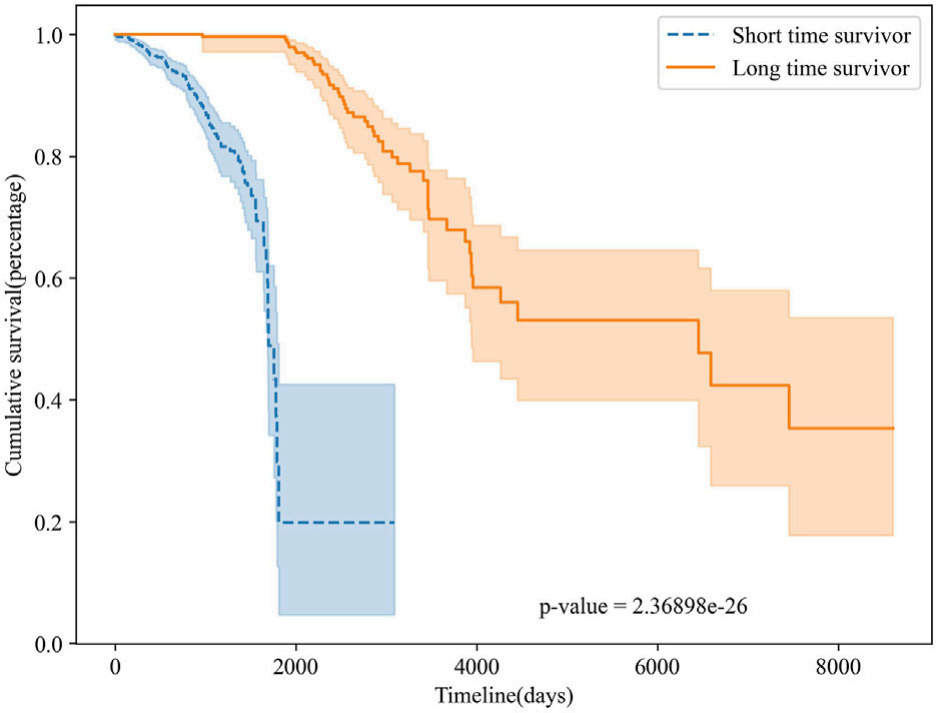
The Kaplan-Meier curves for MSFN.

To further validate the survival prediction performance of MSFN, we performed survival analyses on the classification results of MSFN. We plotted Kaplan-Meier curves to evaluate the performance of MSFN in predicting long-term and short-term survivors Rich et al. (2010), illustrated in Figure 6. The Kaplan-Meier survival curves explicitly demonstrated a statistically significant difference (*p*-value <10e-26) between long-term and short-term survivors predicted by MSFN. This result proves that MSFN effectively distinguishes between long-term and short-term survivors.

**FIGURE 7:**
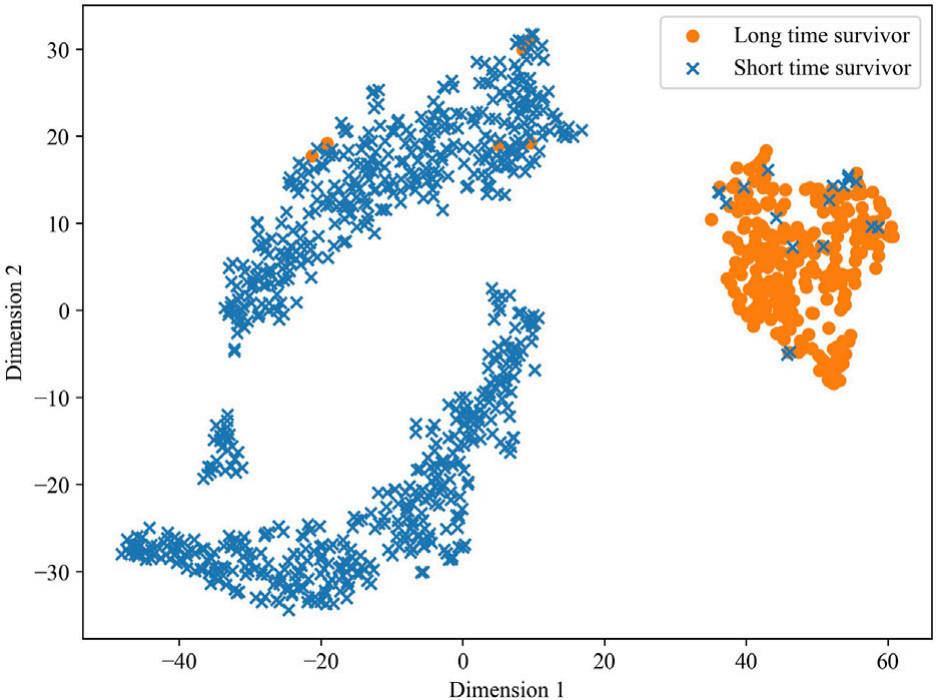
The t-SNE plot of stacked feature classification via MSFN.

To validate the predictive ability of the stacked feature representations obtained in MSFN, we utilized the t-SNE algorithm to visualize the prediction results of the stacked feature representations. t-SNE attempts to minimize the difference between the conditional probabilities or similarities in the high and low dimensional spaces to map the data in the low-dimensional space (Van der Maaten and Hinton, 2008; Wattenberg et al., 2016). The visualization result is shown in Figure 7, a clear demarcation between the two groups at dimension 1 of about 25 indicates the excellent survival prediction ability of the hidden layer features extracted by MSFN.

## 4 Conclusion

Breast cancer is the most prevalent cancer worldwide and poses a major threat to women’s health. Survival prediction can avoid the suffering caused by over-treatment and the waste of medical resources, which is significant for cancer treatment and prognosis. In this study, we propose a novel stacked fusion network (MSFN) for breast cancer survival prediction. MSFN integrates patient similarity, correlation, and specificity information of multi-omics data, providing a more comprehensive insight for survival prediction and effectively enhancing the prediction ability. First, MSFN constructs a patient similarity network and obtains patient similarity information and correlation of multi-omics data through ResGCN. Meanwhile, MSFN obtains the specificity information of multi-omics data through CNN. Finally, MSFN uses the stacking strategy to ingeniously integrate prognostic information and predict patient survival with AdaboostRF. Experiments on TCGA’s breast cancer dataset showed that MSFN outperformed state-of-the-art methods in survival prediction. In future work, we will focus on exploring the survival regression issues. Furthermore, we will explore the interpretability of the survival prediction model to understand the decision-making process of the models and the interpretation of the results.

## Data Availability

The original contributions presented in the study are included in the article/supplementary material, further inquiries can be directed to the corresponding author.
